# Hypersensitivity Pneumonitis-like Granulomatous Lung Disease with Nontuberculous Mycobacteria from Exposure to Hot Water Aerosols

**DOI:** 10.1289/ehp.9542

**Published:** 2006-11-06

**Authors:** Akshay Sood, Rajgopal Sreedhar, Pradeep Kulkarni, Abdur Ray Nawoor

**Affiliations:** 1 University of New Mexico Health Sciences Center, Albuquerque, New Mexico, USA; 2 Southern Illinois University School of Medicine, Springfield, Illinois, USA; 3 Central Illinois Allergy and Respiratory Services Ltd., Springfield, Illinois, USA; 4 Physicians Group Associates, S.C., Springfield, Illinois, USA

**Keywords:** aerosol, granulomatous lung disease, hypersensitivity pneumonitis, hot tub, *Mycobacterium avium* complex, nontuberculous mycobacteria

## Abstract

**Objective:**

Human activities associated with aerosol-generating hot water sources are increasingly popular. Recently, a hypersensitivity pneumonitis (HP)-like granulomatous lung disease, with non-tuberculous mycobacteria from exposure to hot water aerosols from hot tubs/spas, showers, and indoor swimming pools, has been described in immunocompetent individuals (also called “hot tub lung”). Our objective in this study was to examine four additional cases of hot tub lung and compare these cases with others reported in the English print literature on this disease.

**Data sources and extraction:**

We retrospectively reviewed all cases (*n* = 4) of presumptively diagnosed hot tub lung in immunocompetent individuals at the various physician practices in Springfield, Illinois, during 2001–2005. In addition, we searched MEDLINE for cases of hot tub lung described in the literature.

**Data synthesis:**

We summarized the clinical presentation and investigations of four presumptive cases and reviewed previously reported cases of hot tub lung.

**Conclusions:**

There is a debate in the literature whether hot tub lung is an HP or a direct infection of the lung by nontuberculous mycobacteria. Primary prevention of this disease relies on ventilation and good use practices. Secondary prevention of this disease requires education of both the general public and clinicians to allow for the early diagnosis of this disease.

Nontuberculous mycobacteria (NTM) are ubiquitous in the environment (Covert et al. 1999; Dawson 1971; Falkinham 2002; Falkinham et al. 1980) and have a predilection for water supply and collection systems (Collins et al. 1984; Covert et al. 1999; Falkinham 2002, 2003; Falkinham et al. 1980; von Reyn et al. 1993, 1994, 2002). Hot water systems may have even higher numbers of NTM than the source water (du Moulin et al. 1988). Human beings are regularly exposed to these waters, which represent a potential source of infection. Pulmonary disease due to NTM may take on a variety of clinicopathologic presentations, including cavitary disease, diffuse nodular disease, and interstitial disease. Recently, hypersensitivity pneumonitis (HP)-like granulomatous lung disease with NTM from exposure to hot water aerosols from hot tubs/spa pools, showers, and swimming pools has been described in immunocompetent individuals (also called “hot tub lung,” because the greatest number of cases are associated with hot tubs) (Aksamit 2003; Cappelluti et al. 2003; Embil et al. 1997; Grimes et al. 2001; Hanak et al. 2006; Kahana et al. 1997; Khoor et al. 2001; Koschel et al. 2006; Lumb et al. 2004; Mangione et al. 2001; Marchetti et al. 2004; Marras et al. 2005; Mery and Horan 2002; O’Neil et al. 2006; Pham et al. 2003; Rickman et al. 2002; Rose et al. 2000; Scully et al. 1997; Systrom and Wittram 2005; Travaline and Kelsen 2003). Given the increasing popularity of hot tubs in the United States (with > 400,000 hot tubs estimated to have been sold in the year 2000), increased physician and consumer awareness of this disease is warranted. In this article, we describe four additional cases of hot tub lung and review the English print literature on this disease.

## Materials and Methods

We retrospectively reviewed medical records of all cases diagnosed with HP-like granulomatous lung disease with NTM from exposure to hot water aerosols from hot tubs/spa pools, whirlpools, showers, and swimming pools in immunocompetent individuals at the various physician practices in Springfield, Illinois, during the period 2001–2005 (*n* = 4).

In addition, we searched PubMed (National Library of Medicine, Bethesda, MD; available at http://www.ncbi.nlm.nih.gov/entrez/query.fcgi) for cases of hot tub lung described in the English print literature using three sets of queries. The first set of queries included the terms “mycobacterium” and either “hypersensitivity pneumonitis” (47 citations), “alveolitis” (46 citations), or “extrinsic allergic alveolitis” (34 citations). The second set of queries included the terms “mycobacterium” and either “hot tub” (18 citations), “whirlpool” (5 citations), “swimming pool” (86 citations), “spa” (19 citations), or “shower” (8 citations). The third set of queries included only the term “hot tub lung” (15 citations). This search strategy yielded a total of 263 citations of published articles, including several duplicates. We reviewed the abstracts of resulting citations, and selected citations were retrieved for full review (Aksamit 2003; Cappelluti et al. 2003; Embil et al. 1997; Grimes et al. 2001; Hanak et al. 2006; Kahana et al. 1997; Khoor et al. 2001; Lumb et al. 2004; Mangione et al. 2001; Marchetti et al. 2004; Marras et al. 2005; Mery and Horan 2002; O’Neil et al. 2006; Pham et al. 2003; Rickman et al. 2002; Scully et al. 1997; Systrom and Wittram 2005; Travaline and Kelsen 2003). Cases published more than once were treated together (Aksamit 2003; Hanak et al. 2006; Rickman et al. 2002). We searched reference lists of all retrieved articles for additional reports. This revealed an abstract, but because it was not peer-reviewed, we did not include the reported cases in our study (Rose et al. 2000). We also did not include two citations in the Japanese literature and one in the German literature (Kenmotsu et al. 2005; Koschel et al. 2006; Ohashi et al. 2006). We defined poor use practices of hot tubs/spas by the frequency of change of filters (monthly) and of the drainage and refilling of water (quarterly), and also by inadequate decontamination or disinfection, as reported in each article.

Permission for the study was obtained from the local institutional review board. The subject’s informed consent was not considered necessary, given the nature of our research.

## Results

The clinical presentation and investigations of our four cases are summarized in Tables 1 and 2, respectively. These four cases involved exposures from indoor hot tubs. All cases were middle-aged women with subacute presentation of respiratory complaints, with dyspnea and cough being the most common complaints; two of the women also described an initial influenza-like illness. Of the four cases, two were nonsmokers, one was an ex-smoker (8 pack-years) who quit 26 years before disease presentation, and one was a current smoker (28 pack-years). Pulmonary function tests predominantly showed a restrictive physiology. Chest radiography showed diffuse interstitial or nodular opacities in all cases. High-resolution computerized tomography (HRCT) of the chest showed diffuse ground glass opacities in all cases and nodules in three of the four cases. Results of bronchoalveolar lavage fluid (BALF) showed a modest lymphocytic predominance in all cases, with a high CD4/CD8 ratio in one case. Culture of BALF grew *Mycobacterium avium* complex (MAC) in all cases. The histopathologic findings on transbronchial biopsy (TBB) were diagnostic in all four cases, showing well-formed and exuberant nonnecrotizing granulomas with centrilobular and bronchiolocentric distribution. A presumptive diagnosis of hot tub lung was established in all cases. Although the hot tub water was not sampled in any case (and hence a definitive diagnosis could not be established), the clinical presentation appeared to be consistent with this disease. All cases showed resolution of disease: one was treated with abstinence alone, another with additional corticosteroids, and the remaining two were treated with a combination of abstinence, corticosteroids, and antimycobacterial therapy. The duration of corticosteroid treatment was short: 3 weeks for case 4, 4 weeks for case 2, and 6 weeks for case 1. The initial oral dose varied between 30 and 60 mg daily and was subsequently tapered off rapidly. The duration of antimyco-bacterial therapy included 6 months of rifabutin, azithromycin, and ethambutol for case 1, and 9 months of levafloxacin and clarithromycin (with an additional 7 weeks of initial therapy with rifabutin) for case 4. Because each case was treated by a different pulmonologist, the variation in treatment regimens reflected the treating physicians’ preference. Follow-up sputum cultures obtained in case 4 at the conclusion of antimycobacterial therapy did not grow mycobacteria. A review of cases of hot tub lung in the English print literature is also presented in Tables 1 and 2.

## Discussion

### Epidemiology

The incidence of infection by environmental NTM in humans is on the rise, with the number of isolates of NTM exceeding those of *Mycobacterium tuberculosis* in the United States (American Thoracic Society 1997; Falkinham 2002). There are multiple factors associated with this rise, including increased awareness of these microbes as human pathogens; improved methods of detection and culture; increased proportion of the population that is either aging or immunosuppressed; the ubiquitous presence of these organisms in water, biofilms, soil, and aerosols; increased exposure to heated water in daily lives; and increased selection of mycobacteria by certain human activities such as widespread use of disinfectants to which NTM are usually resistant (Falkinham 2002).

Aerosolized environmental NTM enter into the lung (Parker et al. 1983; Wendt et al. 1980) and cause HP-like granulomatous lung disease in machine tool operators exposed to metalworking fluids [Centers for Disease Control and Prevention (CDC) 2002; Kreiss and Cox-Ganser 1997; Moore et al. 2000; Shelton et al. 1999] and in users of aerosol-generating hot water sources (hot tub lung) (Aksamit 2003; Cappelluti et al. 2003; Embil et al. 1997; Grimes et al. 2001; Hanak et al. 2006; Kahana et al. 1997; Khoor et al. 2001; Koschel et al. 2006; Lumb et al. 2004; Mangione et al. 2001; Marchetti et al. 2004; Marras et al. 2005; Mery and Horan 2002; O’Neil et al. 2006; Pham et al. 2003; Rickman et al. 2002; Rose et al. 2000; Scully et al. 1997; Systrom and Wittram 2005; Travaline and Kelsen 2003). Although most cases of hot tub lung are related to hot tub/spa exposure, showers have been shown to be important in one reported case of this disease (Marras et al. 2005). It is also likely that some cases of sauna-takers lung, lifeguard lung, humidifier lung, and tap water–associated HP described in the literature more than a decade ago may, in fact, be unsuspected cases of HP from aerosolized mycobacteria. If true, then hot tub lung is, in fact, not a new disease but a better characterization of a previously described disease.

In several cases of hot tub lung, the source of infection has been proven by genotypic linkage by multilocus enzyme electrophoresis and restriction fragment length polymorphism analysis of the NTM isolated from the source and from the human specimen (Kahana et al. 1997; Lumb et al. 2004; Mangione et al. 2001; Marras et al. 2005). MAC is the most frequently reported NTM associated with hot tub lung, but cases of this disease related to *Mycobacterium fortuitum* have also been reported (Khoor et al. 2001; Mangione et al. 2001).

The growth of MAC is not inhibited by temperatures as high as 42°C—above the usual hot tub temperature (Archuleta et al. 2002). MAC is also approximately 1,000 times moreresistant to chlorine than is *Escherichia coli,* the standard for drinking water disinfection (Taylor et al. 2000). The combination of poor hot tub maintenance, poor personal hygiene (such as entering a hot tub without a prior shower) (Embil et al. 1997; Mangione et al. 2001), uninhibited growth of MAC organisms, and jet aerosolization and subsequent inhalation of large amounts of MAC presumably lead to the development of this disease. Although publication bias prevents a definite conclusion, it should be noted that, of the 16 cases in which data are available, 15 possibly demonstrated “unhygienic” host practices or “faulty” maintenance of hot tubs/spas, including inadequate decontamination or disinfection. Only four cases of hot tub lung have been reported with outdoor hot tub/spa use (Lumb et al. 2004; Travaline and Kelsen 2003). This may be because of the dilution in concentration of mycobacteria in aerosols as a result of better ventilation or because of the mycobactericidal effect of ultraviolet light outdoors.

### Pathogenesis

The pathogenesis of hot tub lung is poorly understood. It is likely that MAC is processed by pulmonary macrophages to T lymphocytes, resulting in their clonal expansion and proliferation, in turn resulting in immunologic responses that induce granuloma formation.

This disease appears to satisfy most clinical criteria for HP outlined in Table 3 (Lacasse et al. 2003)—the gold standard criteria for the diagnosis; that is, the presence of both BALF lymphocytosis and typical HRCT findings are seen in all cases. However, a direct infection of the lung by NTM has been suggested as well, although this subject is still a matter of debate (Aksamit 2003).

The following factors appear to favor the immunologic hypothesis regarding the pathogenesis of hot tub lung. Hypersensitivity reactions have been previously reported to the antigenic components of the acid-rich cell wall of mycobacteria (Molina et al. 1992; Richerson et al. 1982). The intravesical instillation of *Bacillus Calmette-Guerin* for bladder cancer treatment has been complicated by a similar lung disease that is associated with lymphocytic-predominant BALF and absence of mycobacterial growth from respiratory cultures (Israel-Biet et al. 1987; Molina et al. 1992). A similar lung condition has been described in association with *Mycobacterium immunogenum* from exposure to metalworking fluids in machinists (CDC 2002; Moore et al. 2000; Shelton et al. 1999). *M. immunogenum* induces HP in mice as well (Gordon et al. 2006; Thorne et al. 2006), an effect augmented by endotoxin coexposure (Thorne et al. 2006). Gordon et al. (2006) found that observed immunologic changes in the lung were significantly greater in C57Bl/6, 129, and BALB/c mice than in other strains, suggesting that genetic factors may contribute to the susceptibility of workers exposed to *M. immunogenum*–contaminated metalworking fluid aerosols. Further, the clinical and radiographic presentation of hot tub lung is consistent with HP. This includes its occurrence in previously healthy hosts and occasionally acutely, soon after hot tub use. In addition, this disease responds clinically to cessation of exposure and to systemic corticosteroids (rather than worsening with the latter, as would be the case with infections). In addition, clinical response to antimycobacterial therapy is much more rapid than that seen in typical cases of pulmonary MAC disease that requires prolonged multidrug treatment of more than a year.

However, several aspects of this disease are not classical for HP and favor the infectious hypothesis. For instance, the well-formed and occasionally necrotic granulomas with palisaded and multinucleated histiocytes, which overshadow interstitial inflammation (Agarwal and Nath 2006), suggests a response different from that seen in other examples of HP. Also, the elevated CD4/CD8 ratio seen in the BALF in hot tub lung is not typical for HP, although there are reports of variable or increased ratios described with farmer’s lung (Ando et al. 1991; Cormier et al. 1987), lifeguard lung (Rose et al. 1998), and HP of mixed etiology (Costabel et al. 1984) and that related to intravesical instillation of *Bacillus Calmette-Guerin* (Israel-Biet et al. 1987). Further, a preponderance of obstructive physiology is seen in this disease, rather than the restrictive physiology classically seen in most cases of HP (Selman 2003). This is likely due to the exuberant peribronchiolar granulomatous response. This peribronchiolar granulomatous response is also likely responsible for the unusually high specificity of transbronchial biopsies in hot tub lung, as opposed to other examples of HP (Lacasse et al. 2003). Serum precipitins to the offending antigen, a significant predictor of HP (Lacasse et al. 2003; Schuyler and Cormier 1997), are not demonstrable in this disease (Mery and Horan 2002), although precipitins against *M. immunogenum* have been reported in some cases of HP related to metalworking fluids (CDC 2002; Shelton et al. 1999). Finally, the co-occurrence of peripheral tree-in-bud appearance on HRCT scan of the chest consistent with MAC infection centered around small airways (Scully et al. 1997), the isolation of MAC on respiratory culture, and the rare isolation of MAC from blood culture (Khoor et al. 2001) are all suggestive of an infectious rather than an immunologic origin of this disease.

### Descriptive characteristics

The clinical presentation and investigations reported with hot tub lung are summarized in Tables 1 and 2, and the salient features are highlighted below. Although patients with this disease usually have a subacute presentation, there is a significant variation in latency, severity, and temporal course. Patients often do not associate their symptoms with the hot tub exposure, and often further increase their exposure to seek relief of symptoms. Chest radiography shows diffuse infiltrates with both upper and lower lobe predominance described, although a normal chest radiograph does not rule out the diagnosis. HRCT scan of the chest shows diffuse ground glass opacities and nodules. These nodules are centrilobular, ground glass, ill-defined micronodules scattered throughout both lungs. A “normal” HRCT scan virtually rules out the diagnosis. Even though sputum culture is positive for MAC in about 74% of the patients, the addition of TBB and BALF cultures may increase the yield further. Hot tub water usually grows MAC, although the microbiological test needed to analyze *Mycobacterium* is available only in select research and reference laboratories (such as Biosan Laboratories Inc., Warren, MI). The histopathologic findings reveal well-formed and exuberant nonnecrotizing granulomas with centrilobular and bronchiolocentric distribution in most cases (Cheung et al. 2003). This contrasts with sarcoidosis, where the granulomas are predominantly along the lymphatic channels and involve the pleura, interlobular septa, and bronchovascular bundles (Agarwal and Nath 2006; Cheung et al. 2003). Many patients are misdiagnosed with sarcoidosis on initial presentation, although hilar and mediastinal lymphadenopathy is not a prominent radiologic feature of hot tub lung.

### Treatment

As a result of confusion regarding the pathogenesis of hot tub lung, there exists no standard approach to the treatment of the disease. The literature describes significant improvement with mere abstinence from hot tubs. The continued use of hot tubs after decontamination and cleaning is not appropriate in these patients because MAC strains may persist in hot tubs even after decontamination and cleaning several times (Travaline and Kelsen 2003). Oral corticosteroids are probably the next line of treatment; if it fails, anti-mycobacterial therapy or both may be used (Table 2), although the duration of therapy is not known. Current data do not allow for the identification of the subgroup that would benefit from antimycobacterial therapy. The presence of necrotic granulomas may intuitively favor such an approach, although the available data are inadequate to support this hypothesis. Some experts recommend consideration of a combination of antimycobacterial therapy and systemic corticosteroids in moderate to severe cases of this disease (Marras et al. 2005). The proponents of this approach cite the instance of *Pneumocystis jiroveci* (previously called *Pneumocystis carinii*) pneumonia, severe cases of which are also associated with a close coupling of infection and inflammation and are treated with both an antibiotic and corticosteroid (Aksamit 2003). The expected course of hot tub lung, following the above measures, is recovery without relapse (Aksamit 2003). No deaths have been reported from this condition, regardless of the delay in diagnosis or severity of disease at the time of diagnosis.

### Prevention

Several measures, which need to be validated in future studies, may be helpful in the primary prevention of this disease:

- Good ventilation of hot tub room
- Frequent cleaning of hot tub
- Frequent change of hot tub water
- Frequent change of tub water filter
- Superheating the tub water to 70°C for 1 hr before use
- Showering before getting into the hot tub
- Use of disinfectants such as bromine and ultraviolet light.

There are currently no standard guidelines for the maintenance of hot tubs. The role of chlorine, chloramine, chlorine dioxide, ozone, and multiple other disinfectants and detergents in the hot tub setting is minimal, given the resistance of MAC to these agents. This may be related to the presence of an efflux protein that mediates efflux of antibiotics and chemicals from mycobacterial cells (Doran et al. 1997). Therefore, we recommend a greater reliance on ventilation and good use practices rather than disinfectants. Secondary prevention of hot tub lung requires education of both the general public and clinicians to allow for the early diagnosis of this disease. Given the prevalent use of hot tubs, all patients who present with respiratory and constitutional symptoms of unclear origin should be asked about hot tub exposure by their health care providers (Schaefer et al. 2003). Providers need to be aware that even in the absence of a history of exposure to hot tubs/spas, NTM exposure from shower heads, swimming pools, whirlpools, and other aerosol-generating hot water sources may rarely result in a similar disease picture. In addition, all patients should be told of potential risks associated with hot tubs and should be advised appropriate maintenance and use of hot tubs.

## Conclusions

A recently reported illness of HP-like granulomatous lung disease from NTM from exposure to hot water aerosols has been described in immunocompetent individuals (hot tub lung). It is however possible that hot tub lung is not a new disease but a better characterization of a previously described disease. Both infectious and immunologic hypotheses have been advanced to describe the pathogenesis of this disease. It is likely that hot tub lung, as a disease entity in general and specifally due to MAC, is an underappreciated and under-diagnosed entity (Schaefer et al. 2003). Given the increasing popularity of human activities associated with aerosol-generating hot water sources in the United States, increased physician and consumer awareness of this disease is warranted.

## Figures and Tables

**Table 1:** Clinical features of hot tub lung.

Clinical features	Case 1	Case 2	Case 3	Case 4	Review of literature (*n* = 55)^a^
Age (years)	46	49	47	55	43.1 (range, 9–69)
Sex	F	F	F	F	29/55 women (52.7%)
Indoor hot tub exposure	+	+	+	+	38/55 (69.1%), shower in 1/55 (1.8%), outdoor tub/spa in 4/55 (9.1%), and unknown location of hot tub in 11/55 (20%)
Duration of hot tub exposure (in years)	0.7	0.3	1.25	1.5	Range, 1 month to 10 years
Duration of symptoms at presentation (in months)	3	1	5	10	Range, hours to 6 months (mean duration, 2 months)
Poor use practice	−	NR	−	NR	15/16 cases (93.8%)
Smoking history					
Current smoker	+	−	−	−	3/32 cases (9.4%)
Ex-smoker	−	−	−	+	10/32 cases (31.3%)
Nonsmoker	−	+	+	−	19/32 cases (59.4%)
Symptoms					
Dyspnea	+	+	+	+	54/55 cases (98.2%)
Cough	+	+	+	+	45/55 cases (81.8%)
Fever	+	−	−	+	30/55 cases (54.5%)
Chest discomfort	+	+	−	−	14/55 cases (25.5%)
Wheezing	−	−	+	−	4/55 cases (7.3%)
Weight loss	+	−	−	−	11/55 cases (20.0%)
Signs					
Bilateral crackles	−	−	−	−	17/36 cases (47.2%)
Wheezing	−	−	−	+	1/36 cases (2.8%)
Digital clubbing	−	−	−	−	0/36 cases (0%)
Treatment modality					
Corticosteroids and abstinence	−	+	−	−	19/55 cases (34.5%)
Antimycobacterial therapy and abstinence	−	−	−	−	11/55 cases (20.0%)
Corticosteroids, antimycobacterial therapy, and abstinence	+	−	−	+	13/55 cases (23.6%)
Abstinence alone	−	−	+	−	12/55 cases (21.8%)
Response to treatment					
Resolution of disease	+	+	+	+	38/53 cases (71.7%)
Partial resolution of disease	−	−	−	−	15/53 cases (28.3%)
No change or worsening	−	−	−	−	0/53 cases
Alternative diagnosis entertained					
Infection	−	+	+	−	4/26 cases (15.4%)
Sarcoidosis	+	−	−	+	6/26 cases (23.1%)

Abbreviations: −, no; +, yes; F, female; NR, not reported. Not all data were reported for each patient.

a Adapted from multiple references as noted in the text.

**Table 2:** Results of clinical investigations for hot tub lung.

Clinical investigations	Case 1	Case 2	Case 3	Case 4	Review of literature (*n* = 55)^a^
Hypoxemia	−	−	−	−	21/41 cases (51.2%)
Elevated ACE level	−	−	−	ND	4/11 cases (36.4%)
Serum precipitins to mycobacteria	ND	ND	ND	ND	Reported negative in only one case (Mery and Horan 2002)
Chest radiography					
Diffuse interstitial or nodular opacities	+	+	+	+	39/48 cases (81.3%)
Focal and other abnormalities	−	−	−	−	2/48 cases (4.2%)
Normal	−	−	−	−	6/48 cases (12.5%)
High resolution computerized tomography scan					
Ground-glass opacities	+	+	+	+	28/40 cases (70.0%)
Disseminated nodules	+	−	+	+	24/40 cases (60.0%)
Air trapping	−	−	−	−	15/40 cases (37.5%)
Normal	−	−	−	−	0/40 cases (0%)
Pulmonary function test					
Obstructive physiology	−	−	+	−	11/32 cases (34.4%)
Restrictive physiology	+	−	−	+	5/32 cases (15.6%)
Mixed picture or nonspecific abnormality	−	+	−	−	8/32 cases (25.0%)
Isolated reduction in diffusing capacity	−	−	−	−	5/32 cases (15.6%)
Normal	−	−	−	−	3/32 cases (9.4%)
BALF cytology					
Percent lymphocytes	40	ND	ND	36	Mean, 55.6% in 8 cases
CD4/CD8 ratio	ND	ND	ND	6:1	Mean, 11:1 in 7 cases
Histopathology of lung^b^					
Diagnostic TBB	+	+	+	+	17/21 cases (80.9%)
Diagnostic surgical biopsy	ND	ND	ND	ND	15/15 cases (100%)
Well-formed nonnecrotizing granulomas	+	+	+	+	37/41 cases (90.2%)
Necrotizing granulomas	−	−	−	−	3/41 cases (7.3%)
Organizing pneumonia	−	−	−	−	5/41 cases (12.2%)
Patchy interstitial pneumonitis	−	−	−	−	8/41 cases (19.5%)
Mycobacterial microbiology (positive)					
Sputum culture	ND	ND	ND	−	20/27 cases (74.1%)
BALF culture	+ (MAC)	+ (MAC)	+ (MAC)	+ (MAC)	10/16 cases (62.5%)
Lung biopsy stain	ND	ND	ND	ND	7/27 cases (25.9%)
Lung biopsy culture	ND	ND	ND	ND	12/14 cases (85.7%)
Hot tub/source culture	ND	ND	ND	ND	36/38 cases (94.7%)

Abbreviations: −, no; +, yes; ACE, angiotensin-converting enzyme; ND, not done; NR, not reported. Not all data were reported for each patient.

a Adapted from multiple references as noted in the text.

b Of the 41 cases with histopathology reported in the literature, TBB was performed in 21 cases, surgical biopsy in 15, both TBB and surgical biopsy in 5, and an unidentified biopsy type was performed in 10 cases.

**Table 3:** Criteria for classifying hot tub lung as HP (Lacasse et al. 2003).

Criteria for diagnosis of HP	Comments related to hot tub lung
Significant clinical predictors	
Evidence of exposure to a known offending antigen	History of hot tub/spa pool/shower exposure in all patients (100%); isolation of antigen in sputum (74.1%); BALF (62.5%); lung biopsy (85.7%); hot tub/source (94.7%)
Positive precipitating antibodies	Not identified in serum and/or BALF
Recurrent episodes of symptoms	Described with recurrent exposures (Cappelluti et al. 2003; Embil et al. 1997)
Inspiratory crackles	Described in 17/36 cases (47.2%)
Symptoms 4–8 hr after exposure	Usually subacute presentation, acute onset of symptoms after exposure described in a minority (Embil et al. 1997)
Weight loss	Described in 20% cases
Gold standard for accepting diagnosis without additional procedures	
Presence of both BALF lymphocytosis^a^ and bilateral ground glass or poorly defined centrilobular nodular opacities on HRCT scan of the chest	BALF lymphocytosis^a^ seen in 8/8 cases (100%) and HRCT abnormalities seen in 40/40 cases (100%)
Pathological criteria for accepting the diagnosis	
Presence of chronic inflammatory infiltrates along small airways and interstitium (diffuse), and scattered, small, nonnecrotizing granulomas	Seen in 41/41 cases with reported histopathology as defined (100%)

a BALF lymphocytosis defined as ≥ 30% for nonsmokers and ex-smokers and ≥ 20% for current smokers.
